# Ahsa1 and Hsp90 activity confers more severe craniofacial phenotypes in a zebrafish model of hypoparathyroidism, sensorineural deafness and renal dysplasia (HDR)

**DOI:** 10.1242/dmm.011965

**Published:** 2013-05-29

**Authors:** Kelly Sheehan-Rooney, Mary E. Swartz, Feng Zhao, Dong Liu, Johann K. Eberhart

**Affiliations:** 1 Department of Molecular and Cell and Developmental Biology, Institute for Cellular and Molecular Biology, Patterson 522, University of Texas at Austin, Austin, TX 78713, USA; 2 School of Life Science, Peking University, 5 Summer Palace Road, Beijing 100871, China

## Abstract

The severity of most human birth defects is highly variable. Our ability to diagnose, treat and prevent defects relies on our understanding of this variability. Mutation of the transcription factor GATA3 in humans causes the highly variable hypoparathyroidism, sensorineural deafness and renal dysplasia (HDR) syndrome. Although named for a triad of defects, individuals with HDR can also exhibit craniofacial defects. Through a forward genetic screen for craniofacial mutants, we isolated a zebrafish mutant in which the first cysteine of the second zinc finger of Gata3 is mutated. Because mutation of the homologous cysteine causes HDR in humans, these zebrafish mutants could be a quick and effective animal model for understanding the role of *gata3* in the HDR disease spectrum. We demonstrate that, unexpectedly, the chaperone proteins Ahsa1 and Hsp90 promote severe craniofacial phenotypes in our zebrafish model of HDR syndrome. The strengths of the zebrafish system, including rapid development, genetic tractability and live imaging, make this an important model for variability.

## INTRODUCTION

Mutation of the transcription factor GATA3 in humans causes hypoparathyroidism, sensorineural deafness and renal dysplasia (HDR) syndrome (Bilous et al., 1992; Van Esch et al., 2000), which displays a high degree of phenotypic heterogeneity. Many individuals with the mutation do not display the full HDR triad and across patients the severity of defects varies widely and can include palatal and central nervous system defects (Barakat et al., 1977; Bilous et al., 1992; Ferraris et al., 2009; Fujimoto et al., 1999; Fukami et al., 2011; Hasegawa et al., 1997; Lichtner et al., 2000; Muroya et al., 2001; Van Esch et al., 2000). A variety of GATA3 mutations have been described in humans, with differing effects on the function of GATA3 (Nesbit et al., 2004). However, there are no clear genotype-phenotype correlations for HDR (Adachi et al., 2006; Zahirieh et al., 2005). Rather, there is a substantial amount of intrafamilial variation, which has been suggested to possibly be due to genetic background effects (Fukami et al., 2011; Hernández et al., 2007; Mino et al., 2005; Nakamura et al., 2011; Zahirieh et al., 2005). However, the cause of this variation is still unknown.

The incomplete penetrance and highly variable expressivity of HDR syndrome suggest a level of canalization: that, in many instances, development is robust enough to overcome reductions in GATA3 levels. HSP90 activity associates with canalization of phenotypes and disease resistance across diverse taxa (Aridon et al., 2011; Chen and Wagner, 2012; Gangaraju et al., 2011; Lu et al., 2003; Queitsch et al., 2002; Yeyati et al., 2007). Large bodies of evidence show that HSP90 is involved in numerous cellular activities, including protein folding (Johnson, 2012; Taipale et al., 2010). Because there are several missense mutations that cause HDR syndrome, HSP90 activity is a candidate for regulating some of the variability observed in HDR.

Models to investigate the variability of HDR are lacking, although mouse models for all aspects of HDR have been generated (Duncan et al., 2011; Grigorieva et al., 2010; Haugas et al., 2012; Karis et al., 2001; Lilleväli et al., 2006; Lim et al., 2000; van der Wees et al., 2004). Here, we describe a zebrafish point mutation in *gata3* that is homologous to a mutated site in human HDR (Nesbit et al., 2004). We show that *gata3* mutant zebrafish display the HDR triad and have craniofacial defects, the severity of which vary significantly depending upon genetic background. Furthermore, we provide novel insights into the interplay between *gata3*, Ahsa1, Hsp90 and the generation of variability in zebrafish *gata3* mutant phenotypes.

## RESULTS

In a forward genetic screen for zebrafish craniofacial mutants, we isolated the *b1075* mutant allele. Using PCR-based genetic mapping of linkage to simple sequence length polymorphisms (SSLPs), we found tight linkage to z20450 on linkage group 4, with no crossovers out of 196 meioses, and placed the mutation in an interval between z6977 and z11657, with 2 and 11 crossovers, respectively. Finer mapping positioned *b1075* in an ∼325 kb interval between *1075-11* and *1075-8*, each with one crossover in 552 meioses. This interval contained five predicted genes: *itih2*, *similar to kin, atp5c1*, *taf3* and *gata3*. Sequence analysis uncovered a thymidine to adenosine point mutation within exon 4 of *gata3* (supplementary material Fig. S1A), resulting in a predicted cysteine to serine missense mutation in the zinc ion (Zn^2+^)-coordinating domain of zinc finger 2 (supplementary material Fig. S1B,C). Injection of a *gata3* morpholino phenocopied the *b1075* mutant (supplementary material Fig. S1D–F), validating that *b1075* is a mutant allele of *gata3*. The cysteine that is mutated in *b1075* is homologous to a cysteine that is mutated in some cases of human HDR (Nesbit et al., 2004), suggesting that zebrafish could be a HDR model.

**TRANSLATIONAL IMPACT**

**Clinical issue**

Phenotypic variability is a common feature of congenital malformations (birth defects). Although the genetic underpinnings of a large number of birth defects are beginning to be understood, the mechanisms underlying this clinical variability remain unclear. HDR (hypoparathyroidism, sensorineural deafness, renal dysplasia) syndrome, which is characterized by a variety of craniofacial defects, is an autosomal dominant condition caused by mutations in the gene encoding a human zinc finger transcription factor, GATA3. The syndrome is difficult to treat because of the associated clinical diversity. Studies have revealed no correlation between *GATA3* genotype and disease severity, and there are currently no animal models available to investigate the cause of the inherent variability in symptoms.

**Results**

Using a forward genetic screen for craniofacial mutants, the authors identified a *gata3* mutant in zebrafish. The mutant has a missense mutation that disrupts the first cysteine of the second zinc finger domain of Gata3. Mutation of the homologous cysteine has been shown to cause HDR syndrome in humans. The authors report that zebrafish *gata3* mutants have defects affecting the palatal skeleton, ear, embryonic kidney and the gill buds, which are evolutionarily related to the human parathyroid. They show that the severity of these phenotypes is highly dependent upon genetic background. Interestingly, the authors reveal a role for chaperone proteins Ahsa1 and Hsp90 (heat shock protein 90) in mediating the variability in craniofacial defects, at least partially.

**Implications and future directions**

The work provides a novel, effective animal model for studying clinical variability in HDR and related syndromes. Zebrafish *gata3* mutants survive through organogenesis, providing researchers who are interested in HDR syndrome with a resource for analyzing the complete spectrum of defects within the same embryo. Importantly, the genetic-background-specific phenotypic differences provide a means for understanding the cause of phenotypic variation. The genetic conservation between human and zebrafish combined with the unique genetic tools available for zebrafish manipulation could be leveraged to test the function of human *GATA3* mutations in different zebrafish backgrounds and to characterize the chaperone pathways that seem to regulate the severity of phenotypes caused by *gata3* mutation in the model system.

We characterized *gata3* expression to determine whether the tissues disrupted in HDR expressed *gata3* in zebrafish. Neural crest cells within the fate map region that is destined to become the trabeculae (Eberhart et al., 2006; Swartz et al., 2011) expressed *gata3* (supplementary material Fig. S1G, arrow). In amniotes, the pharyngeal endoderm of the second through to the fourth arch generates the parathyroids (Grigorieva et al., 2010; Okabe and Graham, 2004). Although zebrafish embryos lack parathyroids, by 72 hpf the pharyngeal endoderm expressed *gata3* in zebrafish (supplementary material Fig. S1H). This expression extended from the third arch posteriorly, consistent with the zebrafish expression of gene homologs involved in parathyroid specification in amniotes (Hogan et al., 2004; Okabe and Graham, 2004). The ear and the embryonic kidney (pronephros) both expressed *gata3* at 33 hpf (supplementary material Fig. S1I,J). The corpuscles of Stannius also expressed *gata3* and are functionally related to the parathyroids in regulating Ca^2+^ (supplementary material Fig. S1J). These results show that the evolutionary homologs and functional analogs of the tissues affected in human HDR express *gata3* in zebrafish.

**Fig. 1.**
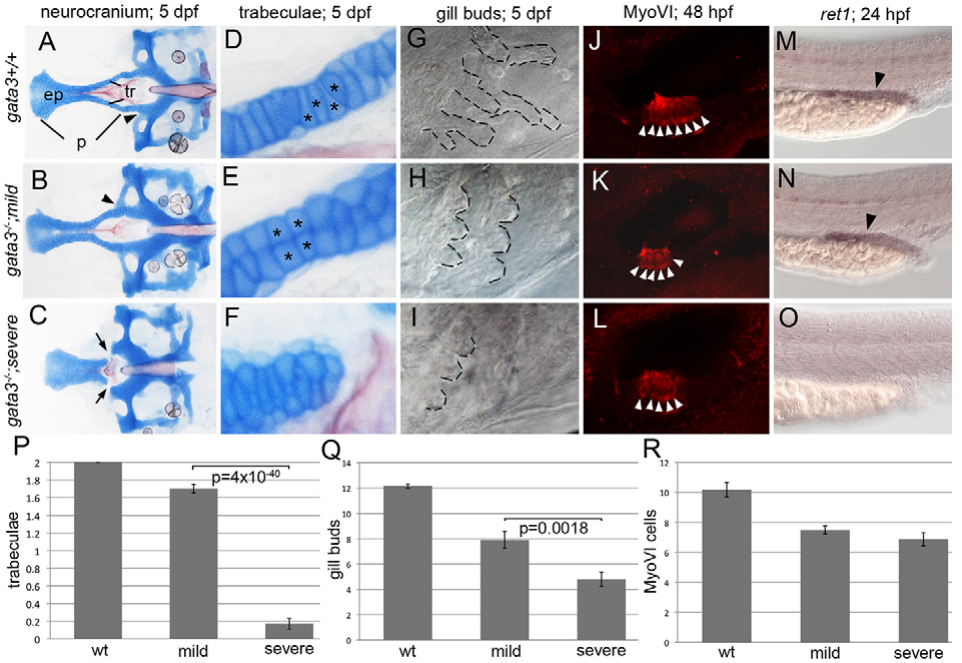
**Genetic background influences the *gata3* mutant phenotypes.** (A–C) Flat-mounted neurocrania and (D–F) close-up views of the trabeculae (tr). In both (A) wild-type embryos and (B) mild *gata3* mutants, the palate (p) is fully formed. However, there are rearrangements to the stacking of chondrocytes in mild mutants (asterisks D and E). (C,F) In severe mutants, the trabeculae are lost, generating a gap between the ethmoid plate (ep) and posterior neurocranium. (G–I) *gata3* mutation disrupts outgrowth of gill buds (outlined). (H) Mild mutants generate fewer gill buds. (I) In severe mutants, the number of gill buds is further reduced. (J–L) Myosin-VI (MyoVI) labels sensory neurons (arrowheads) in the zebrafish ear. (K,L) Mild and severe mutants have fewer MyoVI -positive cells. (M–O) Wild-type and mutant embryos stained with the *ret1* riboprobe. (M,N) *ret1* expression is maintained in mild mutants (arrowheads). (O) In severe mutants, *ret1* expression is absent. (P–R) Quantification of the defects in *gata3* mutant embryos. All graphs show means ± 1 s.e.m. (P) The number of trabeculae per embryo are significantly reduced in severe mutants (average=0.17, s.e.m.=0.06, s.d.=0.46, *n*=58) compared with mild mutants (average=1.7, s.e.m.=0.05, s.d.=0.55, *n*=125). Wild-type embryos average=2 trabeculae (s.e.m.=0, s.d.=0, *n*=654). (Q) The number of gill buds per embryo is also significantly reduced in severe mutants (average=4.79, s.e.m.=0.57, s.d.=2.12, *n*=14) relative to mild mutants (average=7.92, s.e.m.=0.68, s.d.=2.35, *n*=12). Wild-type embryos average=12.15 gill buds (s.e.m.=0.17, s.d.=0.75, *n*=20). (R) Although there is a reduction compared with wild type (average=10.17, s.e.m.=0.49, s.d.=1.70, *n*=11), the number of MyoVI-positive cells are not significantly altered across mild (average=7.5, s.e.m.=0.27, s.d.=0.85, *n*=10) and severe (average=6.88, s.e.m.=0.44, s.d.=1.25, *n*=8) mutants. Anterior to the left; (A–F) dorsal views of flat-mounted neurocrania; (G–O) lateral views of whole-mounted embryos.

To test for functional conservation of GATA3 between zebrafish and human, we examined HDR tissue homologs in our zebrafish mutant. Our analyses were performed across two different genetic backgrounds, WIK and EkkWill, and, although phenotypes across wild-type embryos did not vary (and are combined for statistics), substantial variation was observed in mutants, across backgrounds (Fig. 1). We refer to the WIK and EkkWill backgrounds as the ‘mild’ and ‘severe’ backgrounds, respectively, because WIK mutants were consistently milder. We initiated our analyses on the craniofacial skeleton because this is where we found the greatest degree of variability.

The neurocranium of the zebrafish (Fig. 1A–C) lies immediately ventral to the brain. The anterior neurocranium, or the zebrafish palatal skeleton, is positioned medial to the eyes and consists of a midline ethmoid plate and bilateral trabeculae. In the mild background, the trabeculae were consistently present, whereas, in a severe background, the trabeculae were consistently absent (Fig. 1B,C). Quantification of the number of intact trabeculae/embryo demonstrated that wild type, mild mutants and severe mutants averaged 2 (*n*=654), 1.7 (*n*=125) and 0.17 (*n*=58) trabeculae, respectively (Fig. 1P). The difference in the average number of trabeculae/embryo between mild and severe mutants was extraordinarily significant as determined via Student’s *t*-test (*P*=4×10^−40^). Interestingly, the variation around the mean was similar within both mild and severe mutants, with standard deviation (s.d.)=0.55 and 0.46, respectively. In wild-type embryos, chondrocytes within the trabeculae were stacked one upon another in a largely single-file fashion (Fig. 1D, asterisks). Although the trabeculae were present, chondrocytes fail to stack appropriately in mild mutants (Fig. 1E, asterisks). Additionally, in 100% of mild and severe mutants, the lateral commissure connected to the trabeculae, instead of its normal more posterior position (Fig. 1A,B, arrowheads).

We next analyzed gill buds because they are evolutionarily related to and require the gene regulatory networks involved in parathyroid development in amniotes (Hogan et al., 2004; Zajac and Danks, 2008). Gill bud length was decreased in both mild and severe *gata3* mutants (Fig. 1G–I, outlined). Whereas wild-type embryos averaged 12.2 gill buds (s.d.=0.75, *n*=20), mild and severe mutants averaged 7.9 (s.d.=2.35, *n*=12) and 4.8 (s.d.=2.12, *n*=14), respectively. This difference in gill bud number between mutants was significant (*P*=0.0018).

We stained sensory hair cells within the ear via anti-MyoVI antibodies (Fig. 1J–L) to test for ear defects. The average number of sensory hair cells in wild-type embryos was 10.2 (s.d.=1.70, *n*=12). Although mutants had a reduced number of sensory hair cells relative to wild type, mild and severe mutants did not significantly vary relative to each other, averaging 7.5 (s.d.=0.85, *n*=10) and 6.88 (s.d.=1.25, *n*=8) cells, respectively. We labeled the pronephros with *ret1* (Fig. 1M–O) to test for renal defects. *ret1* was expressed in the pronephros of mild mutants but absent in severe mutants (Fig. 1N,O). Collectively, these data show that, like in human, mutation of *gata3* causes variable defects, making zebrafish a tractable model to understand variability within HDR syndrome. To test our ability to modulate HDR phenotypes, we examined the function of pathways with known involvement in disease variability and canalization.

**Fig. 2.**
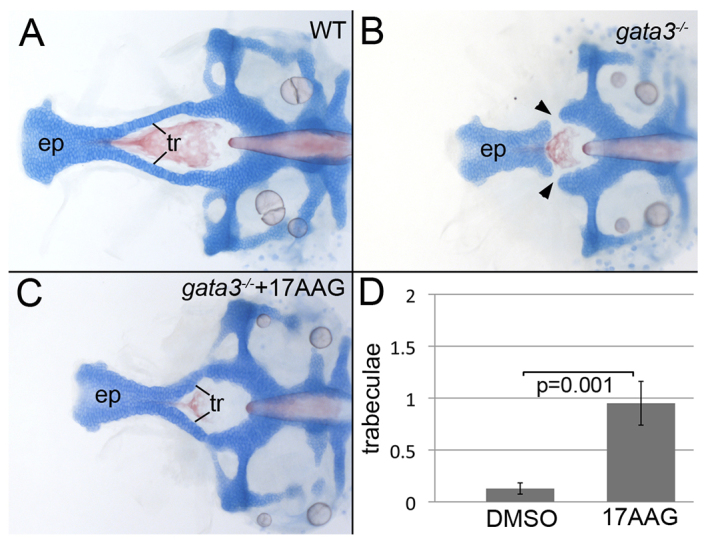
**Inhibition of Hsp90 partially restores trabeculae in severe mutants.** (A) Zebrafish embryos were treated with levels of 17AAG that did not disrupt the craniofacial skeleton in wild type. (B) Untreated *gata3* mutants in the severe background typically lack trabeculae (arrowheads). (C) Hsp90 inhibition partially restored the trabeculae, resulting in the fusion of the palate and posterior neurocranium. (D) 17AAG treatment of severe *gata3* mutants significantly increases the number of trabeculae in treated embryos (average=0.95, s.e.m.=0.211, s.d.=0.94, *n*=20) compared with control, DMSO-treated, embryos (average=0.13, s.e.m.=0.05, s.d.=0.34, *n*=39). (A–C) Dorsal views of flat-mounted neurocrania, anterior to the left; ep, ethmoid plate; tr, trabeculae.

Across a wide range of taxa an important modulator of phenotypic variability and canalization is heat shock protein 90 (HSP90) (Aridon et al., 2011; Chen and Wagner, 2012; Gangaraju et al., 2011; Lu et al., 2003; Queitsch et al., 2002; Yeyati et al., 2007), making HSP90 a promising candidate to modulate HDR phenotypic variability. We focused on the craniofacial phenotype because it is strikingly canalized in the mild background. We treated zebrafish embryos with the HSP90 inhibitor 17AAG to determine whether Hsp90 was involved in the across-background variability. Surprisingly, we found that inhibition of Hsp90 caused the partial restoration of the trabeculae in the severe genetic background (Fig. 2) but did not alter the craniofacial phenotype of mild mutants (data not shown). Hsp90 inhibition did not fully rescue the morphology of the anterior neurocranium, but significantly (*P*=0.001) increased the number of trabeculae that developed and fused to the posterior neurocranium in severe mutants (Fig. 2D). Treated mutants averaged 0.95 trabeculae (s.d.=0.94, *n*=20) and untreated mutants averaged 0.12 trabeculae (s.d.=0.34, *n*=39). This result demonstrates that Hsp90 activity promoted more severe *gata3* mutant phenotypes.

**Fig. 3.**
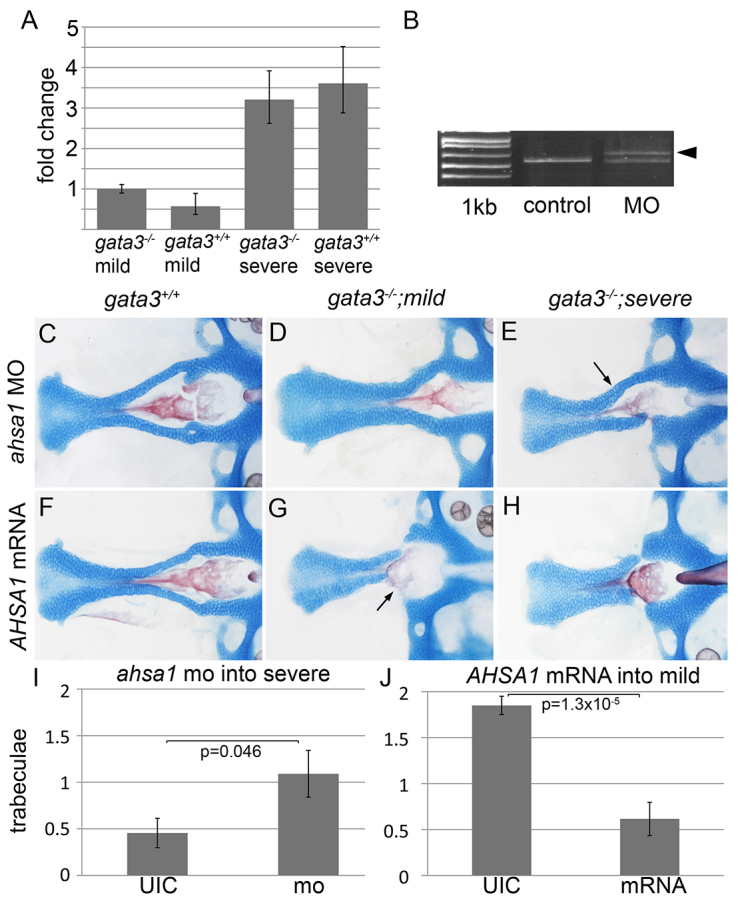
**Ahsa1 is necessary and sufficient to promote severe *gata3* phenotypes.** (A) *ahsa1* expression is higher in *gata3* mutants and wild-type embryos in the severe background. (B) The *ahsa1* morpholino causes misplicing of *ahsa1* mRNA [arrowhead in morpholino (MO) lane; 1 kb=1 kb plus ladder]. (C,D) In both wild-type and mild mutant embryos *ahsa1* morpholino injection did not disrupt the trabeculae. (E) Knocking down *ahsa1* in severe mutants resulted in a partial restoration of the trabeculae (arrow). (F) Wild-type embryos injected with low levels of *AHSA1* mRNA display no craniofacial defects. (G,H) Under these conditions, mutants from the (G) mild background closely resemble those from the (H) severe background, with trabeculae loss (arrows). (I) Ahsa1 loss-of-function significantly increases the number of trabeculae in severe *gata3* mutants (average=1.09, s.e.m.=0.25, s.d.=0.83, *n*=11), relative to uninjected control mutants (average=0.455, s.e.m.=0.16, s.d.=0.52, *n*=11). (J) Injection of *AHSA1* mRNA significantly reduces the number of trabeculae in mild *gata3* mutants (average=0.615, s.e.m.=0.18, s.d.=0.37, *n*=13), relative to uninjected mutants (average=1.85, s.e.m.=0.1, s.d.=0.65, *n*=25). (C–H) Flat-mount images; anterior to the left.

Although most evidence points to Hsp90 buffering against severe phenotypes, evidence in cystic fibrosis suggests that AHSA1-mediated HSP90 activation might correlate with a poorer prognosis (Wang et al., 2006). To determine whether Ahsa1 might be regulating *gata3* mutant phenotypes in zebrafish, we performed qPCR to compare *ahsa1* mRNA levels across genetic backgrounds. In an initial characterization, we found that *ahsa1* was elevated 3.4-fold in severe mutants relative to mild mutants (data not shown). To confirm and extend this finding, we tested *ahsa1* levels in mutants and wild types across both backgrounds. Similarly, we found that *ahsa1* was elevated 3.2-fold in severe mutants relative to mild mutants and that these differences seem to largely be due to differences in expression levels across the genetic backgrounds (Fig. 3A).

To test the functional significance of this expression difference, we injected *ahsa1* splice-site-blocking morpholinos (Fig. 3B) at doses that left wild-type embryos unaffected (Fig. 3C). These levels failed to alter mild mutant phenotypes (Fig. 3D), but improved the phenotype of severe mutants (Fig. 3E,I). Uninjected severe mutants averaged 0.45 trabeculae (s.d.=0.52, *n*=11), whereas *ahsa1*-morpholino-injected severe mutants averaged 1.09 trabeculae (s.d.=0.83, *n*=11, *P*=0.046). This result shows that Ahsa1 is necessary to promote severe craniofacial phenotypes in *gata3* mutants.

To test whether Ahsa1 was sufficient for severe phenotypes, we injected mRNA encoding human AHSA1 at levels that did not affect wild-type embryos (Fig. 3F). We found that *AHSA1* injection caused loss of the trabeculae in mild mutants (Fig. 3G). Uninjected mild mutants averaged 1.85 trabeculae (s.d.=0.35, *n*=25), whereas *AHSA1*-injected mild mutants averaged 0.62 trabeculae (s.d.=0.65, *n*=14; *P*=0.000013; Fig. 3G,J), suggesting that the Ahsa1-Hsp90 pathway is more active in severe mutants.

**Fig. 4.**
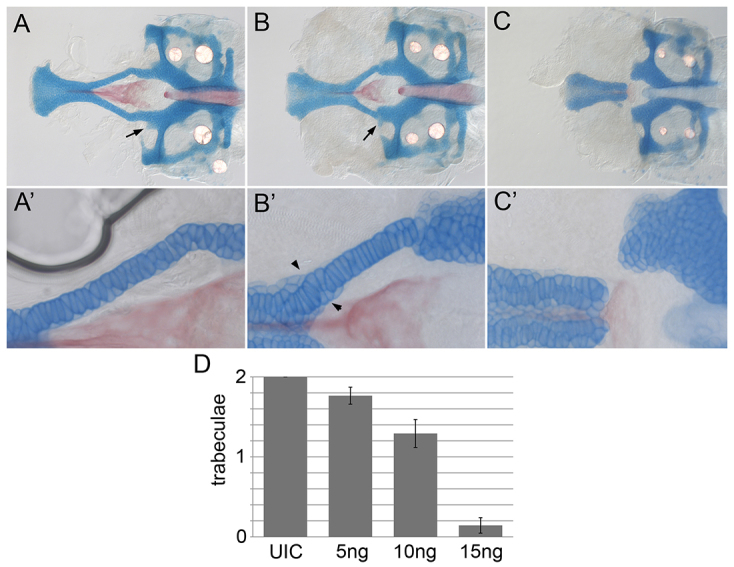
**Graded injection of *gata3* morpholino recapitulates the background-specific *gata3* mutant phenotypes.** (A,A′) Uninjected (UIC) embryos have normal neurocrania and trabeculae. (B,B′) Injection of 5 ng *gata3* morpholino results in the lateral commissure attaching to the trabeculae (arrow, B) and improper stacking of cells within the trabeculae (arrowheads, B′). (C,C′) Injection of 15 ng of *gata3* morpholino causes trabeculae loss. (D) Quantification of the average number of trabeculae/embryo.

Ahsa1-Hsp90 activity sequesters mutant CFTR, thus lowering its effective concentration (Wang et al., 2006). If the effective concentration of Gata3 is different across genetic backgrounds then a graded morpholino injection should recapitulate phenotypes observed in both backgrounds. Indeed, we found that inbred AB embryos injected with 5 ng of a *gata3* morpholino very closely resembled mild mutants, with alteration to the attachment of the lateral commissure (Fig. 4B, arrow) and cell stacking of the trabeculae (Fig. 4B′, arrowheads) but with mostly attached trabeculae (Fig. 4B,B′,D). Embryos injected with 15 ng of morpholino had loss of the trabeculae that closely resembled severe *gata3* mutants (Fig. 4C–D). Collectively, our data demonstrate that, as in human, mutation of *gata3* in zebrafish causes a highly variable set of defects. These data demonstrate that the AHSA1-HSP90 pathway is involved in generating genetic-background-dependent variation in zebrafish *gata3* mutants and suggest that zebrafish will aid in understanding variability of human HDR.

## DISCUSSION

Mutation of *GATA3* causes HDR syndrome in humans. We show that tissues disrupted in HDR are defective in our zebrafish mutants. Our zebrafish mutation is in the homologous residue to human C318. In HDR this cysteine can be replaced by arginine (Nesbit et al., 2004), whereas in our zebrafish model a serine is generated. Although the serine replacement is more conservative, it is still predicted to result in a loss of Zn^2+^ coordination within the second zinc finger domain, which requires the cysteine. Indeed, an in-depth analysis of human GATA3 mutations showed that any disruptions to the second zinc finger domain resulted in a loss of DNA binding (Nesbit et al., 2004). Future experiments will be aimed at determining whether the zebrafish mutation behaves similarly in these types of analyses.

Our results demonstrate that activation of the Hsp90 pathway is deleterious for zebrafish *gata3* mutants. This was surprising given the clear role of Hsp90 in protecting against deleterious phenotypes (Gangaraju et al., 2011; Lu et al., 2003; Queitsch et al., 2002; Yeyati et al., 2007). Although HSP90 seems to function predominantly by stabilizing proteins (Taipale et al., 2010), in cystic fibrosis Ahsa1 downregulation enhances CFTR activity (Wang et al., 2006). The deleterious activity of HSP90 in cystic fibrosis is thought to be due to HSP90 sequestration of a hypomorphic CFTR. Therefore, in our zebrafish model Hsp90 sequestration might block functions that the missense Gata3 protein retains. Although the human GATA3 C318R mutation fails to bind DNA *in vitro* (Nesbit et al., 2004), it is possible that, *in vivo*, higher-order protein complexes compensate for this reduced DNA-binding capacity. Our future analyses will include testing the function and localization of human and mutant forms of GATA3 protein across genetic backgrounds in zebrafish.

The functional role of Gata3 that is buffered in the mild mutants is still not understood. In severe *gata3^b1075^* mutants, neural crest cells that should form the trabeculae become mislocalized (M.E.S. and J.K.E., unpublished). This, coupled with the stacking defect in mild mutants, suggests that Hsp90 activity modulates a role for Gata3 in neural crest cell movements underlying palatogenesis. Analysis of neural crest cell movements across genetic backgrounds will provide important insights into the role of Gata3 in craniofacial development.

## MATERIALS AND METHODS

### Zebrafish care and husbandry

Zebrafish care protocols were IACUC approved and performed according to Westerfield (Westerfield, 1993). 17AAG stock was dissolved in DMSO and applied as described (Yeyati et al., 2007). The *gata3^b1075^* allele was generated through ENU-mediated mutagenesis in a Tubigen background. The same female carrier was crossed to the EkkWill and WIK genetic backgrounds. Genetic mapping was performed in the WIK background. The F1 offspring of these crosses exhibited dramatically different phenotypes (as described) and, through continuous incrossing, these phenotypic differences have consistently been maintained.

Genotyping of *gata3* was performed using primers: jke71 (f: 5′-GGAAACAGAAGGGGATGGGG-3′) and jke72 (r: 5′-TCTTACTAGAGAAGTGTAAGACAGCTAGGG-3′), followed by restriction digestion with *Nla*III: the mutant allele is 273 bp, and the wild-type allele is 138 and 139 bp.

### Staining protocols

5-dpf zebrafish larvae were stained with Alcian Blue and Alizarin Red (Walker and Kimmel, 2007). For *in situ* hybridization and immunohistochemistry, embryos were fixed in 4% PFA between 32 and 72 hpf. The *gata3* and *ret1* riboprobes are described (Neave et al., 1995; Wingert et al., 2007).

A rabbit polyclonal antibody was used at 1:500 to analyze MyoVI protein expression (Proteus Biosciences, 25-6791). Embryos were fixed in 4% PFA overnight at 4°C. Embryos were washed with PBS then water before treating with 100% acetone for 10 minutes at −20°C. Embryos were washed with water then PBS and then incubated in blocking solution, containing 2% normal goat serum in PBDTx (PBS, 1% BSA, 1% DMSO, 0.5% Triton-X), for 1 hour at room temperature. Anti-MyoVI antibody in blocking solution was applied overnight at 4°C. Following washing, a 1:200 dilution of the secondary antibody (Alexa-Fluor-568, Invitrogen) in PBDTx was applied for 5 hours, at room temperature.

All embryos used for skeletal staining or *in situ* hybridization were imaged on a Zeiss Axioimager. Embryos analyzed by immunohistochemistry were imaged on a Zeiss 710 confocal microscope.

### Injections and pharmaceutical treatments

Injections were performed at the one-cell stage. The E4I4 *ahsa1* morpholino (5′-TTAGAGCAGTCACCTGTTTTGAGAT-3′; Gene Tools) targets the splice site between exon4 and intron4. A 3 nl bolus of an 8 mg/ml morpholino solution was injected. The primer pair ksr82 (5′-CCCAGCACAGCTAATGCTCC-3′) with ksr83 (5′-TGCTGGCCAACTAGCAAACC-3′) assessed morpholino efficacy. The translation blocking *gata3* morpholino 5′-CCGGACTTACTTCCATCGTTTATTT-3′ (Gene Tools) (Yang et al., 2010) was used.

Tol2 competent human *AHSA1* (Orfeome, Invitrogen) was cloned into pCSDest (Villefranc et al., 2007) following the manufacturer’s instructions (LR Clonase, Invitrogen). *AHSA1*:pCSDest was linearized with *Apa*I and mRNA was transcribed using the Sp6 mMessage Machine kit (Ambion). A 3 nl injection of a 300 ng/μl stock of *AHSA1* mRNA was injected.

### qPCR and statistical analyses

Student’s *t*-tests were performed between mild mutants and severe mutants. All graphs show the mean ± 1 s.e.m. qPCR was performed in triplicate with Sybr Green (ABI) on a Viia7 system (Invitrogen) according to the manufacturers’ protocols. At 36 hpf, embryos were genotyped using DNA extracted from tails, and heads were stored in RNAlater (Qiagen). Following genotyping, nine heads from *gata3^−/−^* and *gata3^+/+^* embryos from each background were pooled and RNA was extracted in Trizol reagent (Invitrogen) followed by DNase treatment and purification (RNAeasy MinElute Cleanup Kit, Qiagen). *ahsa1* was amplified using primers ksr101 (5′-ACAGAGTTCGCTCAGGGTAT-3′) and ksr87 (5′-GCGCCCATCCACAAAAGCAGC-3′), and normalized to *rpl13a* (Tang et al., 2007).

## Supplementary Material

Supplementary Material
