# Supplementary material for: Ahsa1 and Hsp90 activity confers more severe craniofacial phenotypes in a zebrafish model of hypoparathyroidism, sensorineural deafness and renal dysplasia (HDR)
Source: Dis Model Mech. 2013 May 29;6(5):1285–91. doi: 10.1242/dmm.011965 (PMC3759348; doi:10.1242/dmm.011965)
Supplement: Supplementary Material [file supp_6_5_1285__index.html]

Ahsa1 and Hsp90 activity confers more severe craniofacial phenotypes in a zebrafish model of hypoparathyroidism, sensorineural deafness and renal dysplasia (HDR) — Ahsa1 and Hsp90 activity confers more severe craniofacial phenotypes in a zebrafish model of hypoparathyroidism, sensorineural deafness and renal dysplasia (HDR) — Supplementary Material 

# Ahsa1 and Hsp90 activity confers more severe craniofacial phenotypes in a zebrafish model of hypoparathyroidism, sensorineural deafness and renal dysplasia (HDR)

## 

**Files in this Data Supplement:**

- **Supplementary Material PDF**
