# Supplementary material for: Ahsa1 and Hsp90 activity confers more severe craniofacial phenotypes in a zebrafish model of hypoparathyroidism, sensorineural deafness and renal dysplasia (HDR)
Source: Dis Model Mech. 2013 May 29;6(5):1285–91. doi: 10.1242/dmm.011965 (PMC3759348; doi:10.1242/dmm.011965)
Supplement: Supplementary Material [file supp_011965_DMM011965.pdf]

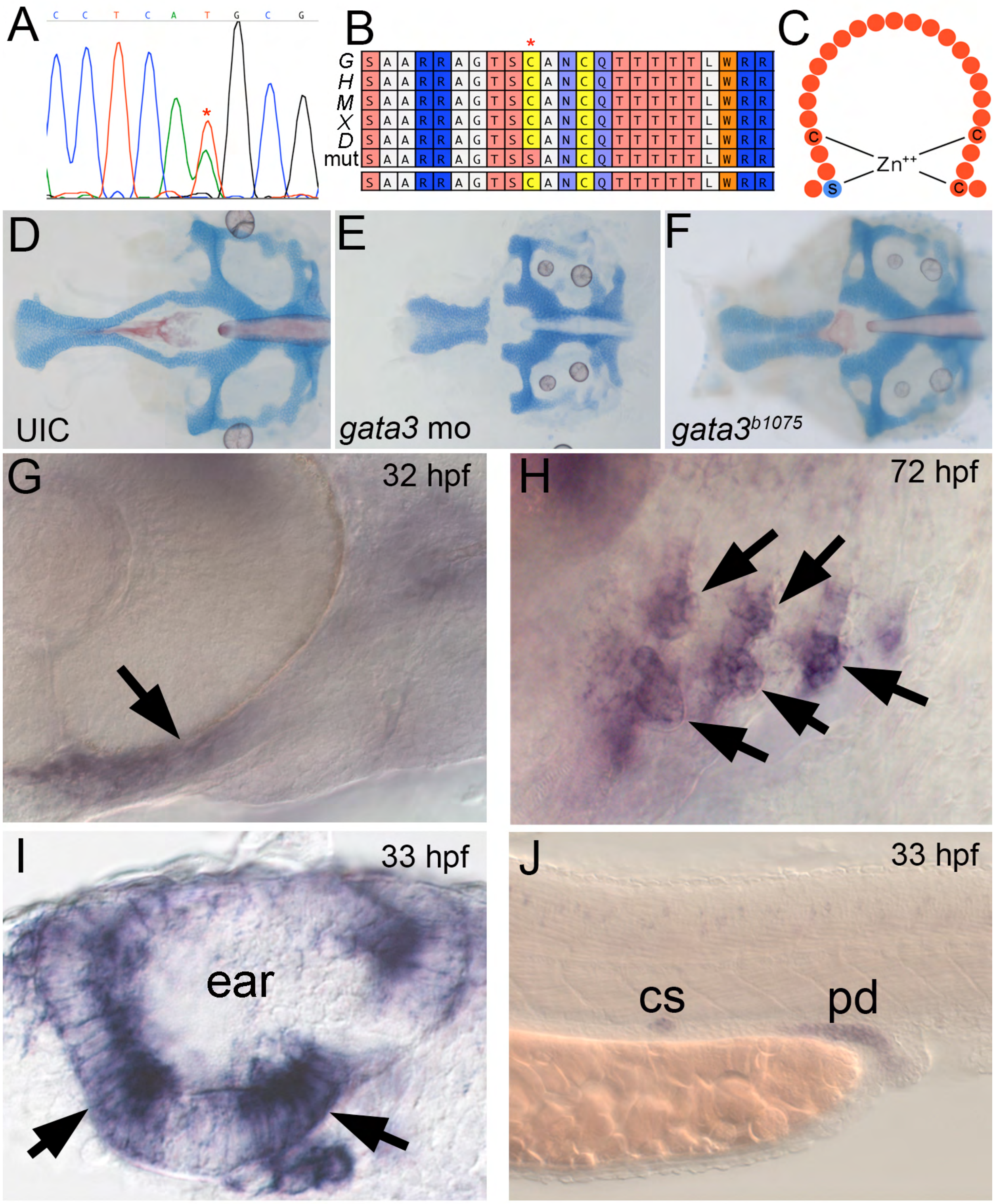

Supplemental Fig. 1. Identification of a *gata3* mutant zebrafish and expression of *gata3*. (A) The *b1075* allele has a T>A point mutation (asterisk) in the 28th bp of the fourth exon of *gata3*. The chromatograph shows a heterozygote. (B) The mutation generates a predicted C>S missense mutation in the conserved 2nd Zinc Finger domain of Gata3 (*G, Gallus gallus; H, Homo Sapien; M, Mus musculus; X, Xenopus laevis; D, Danio rerio, mut, b1075*). (C) Schematic representation of the affected amino acid. (D-F) Embryos (E) injected with *gata3* morpholino phenocopy (F) *b1075* mutants. (G-J) Tissues disrupted in HDR express *gata3*. (G) Maxillary neural crest cells express *gata3* (arrow). (H) Forming gill buds express *gata3* by 72 hpf (arrows). (I) At 33 hpf, *gata3* is expressed strongly in regions of the ear that will generate sensory cells (arrows). (J) Both the pronephric duct (pd) and Corpuscle of Stannius (cs) express *gata3* at 33 hpf. Lateral views, anterior to the left. UIC=uninjected control.
